# Retrospective observational cohort study of mortality and length of stay for surgical ICU admissions

**DOI:** 10.1186/cc14599

**Published:** 2015-03-16

**Authors:** M Hameed, M Maruthappu, D Marshall, M Pimentel, L Celi, J Salciccioli, J Shalhoub

**Affiliations:** 1 University of Oxford, UK; 2 National Institute for Health and Care Excellence, London, UK; 3 Imperial College London, UK; 4 Massachusetts Institute of Technology, Cambridge, MA, USA

## Introduction

A significant number require a postoperative ICU stay which may be prolonged [1]. Very limited data exist to characterise mortality and ICU length of stay (LOS) in different surgical specialties [2,3]. We aim to describe this relationship in a large cohort of surgical patients.

## Methods

We performed a retrospective observational cohort study of adult surgical admissions to the ICU of a large academic tertiary medical centre. The primary endpoint was 28-day mortality. We used simple descriptive statistics to characterise the population. The Wilcoxon rank-sum test or Kruskall-Wallis test was used, as appropriate, for tests of continuous data.

## Results

There were 6,203 surgical admissions during the 8-year study period. For both ICU and in-hospital mortality; the median LOS in days for survivors was 2.2 (IQR: 1.2 to 4.9) and that of nonsurvivors was 3.2 (IQR: 1.5 to 7.9). At 28 days, 1 year, and 2 years, the respective values were 2.2 (1.2 to 4.9) and 3.3 (1.5 to 6.7); 2.1 (1.2 to 4.7) and 3.1 (1.5 to 7.3); and 2.1 (1.2 to 4.6) and 3.0 (1.5 to 7.0), all *P *< 0.0001. The greatest median LOS was found in neurosurgery and cardiothoracic surgery; 3.3 days (IQR: 1.7 to 9.5) and 3.1 days (IQR: 1.5 to 8.0) respectively. They corresponded to the specialities with the greatest percentage ICU (9.7% and 10.2%) and 2-year mortality (27.9%, and 35%). The greatest mortality and median LOS was found in ventriculostomy cases; 40.8% at 2 years and 10.6 days (IQR: 4.8 to 18.2).

## Conclusion

There is an association between postoperative ICU LOS and mortality that persists for at least 2 years after admission. Neurosurgery and cardiothoracic surgery patients appear to have a worse prognosis and also a more prolonged LOS. Our results may provide a more objective basis for clinical decisions, the use of limited resources, and inform on appropriate expectations of treatment.
